# Influence of Centrifugation Cycles of Natural Rubber Latex on Final Properties of Uncrosslinked Deproteinized Natural Rubber

**DOI:** 10.3390/polym14132713

**Published:** 2022-07-02

**Authors:** Nabil Hayeemasae, Sitisaiyidah Saiwari, Siriwat Soontaranon, Abdulhakim Masa

**Affiliations:** 1 Department of Rubber Technology and Polymer Science, Faculty of Science and Technology, Prince of Songkla University, Pattani Campus, Pattani 94000, Thailand; nabil.h@psu.ac.th (N.H.); sitisaiyidah.s@psu.ac.th (S.S.); 2 Research Unit of Advanced Elastomeric Materials and Innovations for BCG Economy (AEMI), Faculty of Science and Technology, Prince of Songkla University, Pattani Campus, Pattani 94000, Thailand; 3 Synchrotron Light Research Institute, Muang District, Nakhon Ratchasima 30000, Thailand; siriwat@slri.or.th; 4 Rubber Engineering & Technology Program, International College, Prince of Songkla University, Hat Yai District, Songkhla 90110, Thailand

**Keywords:** natural rubber, deproteinized natural rubber, mechanical properties, protein content

## Abstract

Natural rubber latex (NRL) is a polymer (blend) extracted from the milky sap of para rubber trees. Due to being a natural biopolymer, NRL contains various proteins that may be allergenic to humans when in skin contact. Attempts have been made to use deproteinized natural rubber (DPNR) instead of impure NRL, and the final properties of these two types of rubber tend to differ. Thus, the correlations between their chemistry and properties are of focal interest in this work. DPNR was prepared by incubating NRL with urea, followed by aqueous washing/centrifugation. The physical, mechanical, and dynamic properties of incubated NRL before and after washing/centrifugation were examined to distinguish its influences from those of incubation with urea. According to the findings, the proteins, phospholipids, and chain entanglements were responsible for natural polymer networks formed in the NR. Although the proteins were largely removed from the latex by incubation, the properties of high ammonia natural rubber (HANR) were still maintained in its DPNR form, showing that other network linkages dominated over those contributed by the proteins. In the incubated latex, the naturally occurring linkages were consistently reduced with the number of wash cycles.

## 1. Introduction

Natural rubber latex (NRL), a milky fluid obtained from the *Hevea brasiliensis* tree, is the key raw material used in producing latex-dipped products like gloves and condoms [1]. It consists of about 25 to 45 wt% rubber hydrocarbon particles dispersed in water, and some minor non-rubber ingredients like proteins, lipids, carbohydrates, sugars, and metal ions [2]. Some of the non-rubber components, in particular the proteins, have been assumed to play essential roles in bringing about the exceptional features of NR: for example, the proteins facilitate natural network formation, boosting the tensile strength and toughness of the NR [3]. Proteins found in the NR latex have recently been related to various allergic reactions in humans. It is believed that the proteins in NRL, particularly type I immunoglobulin E located around the surfaces of the rubber particles, are allergenic [4]. Thus, reducing the amount of protein could broaden the use and applications of NR. Several attempts have been made to bring the protein content in natural rubber products down to an acceptable level. These include the use of low-protein latexes, optimized pre-cure and post-cure leaching techniques, chemical or enzymatic deproteinization, chlorination, and coating [1]. Among the various approaches, chemical or enzymatic deproteinization approaches appear the most promising.

The term “deproteinization” means reducing the amount of proteins, possibly completely removing them. After deproteinization, the obtained latex was called deproteinized NRL (DPNR). After deproteinization, the latex will be called deproteinized NRL (for short DPNR). The process can be enzymatic [5,6], in which case the latex is initially stabilized with a suitable surfactant and treated with a proteolytic enzyme, such as alkaline protease. This technique typically involves centrifugation, either once or twice, to eliminate the degraded proteins. This approach was determined to be the most effective and efficient in removing proteins from NR latex. Various studies have prepared DPNR in this manner and the nitrogen content has been dramatically reduced from the initial around 4% to less than 0.02% [5,7,8,9]. A lengthy incubation period of at least 24 h is required and this is a limitation of the enzymatic approach. In addition, the remaining proteins, peptides, or amino acids may still result in intraoperative anaphylactic reactions in hypersensitive patients with allergies [9]. An alternative deproteinization method has been developed in which the latex is incubated with urea in the presence of a surfactant, followed by washing twice with centrifugation. This method is particularly attractive because the deproteinization could be accomplished in 1 h under suitable conditions, and the total nitrogen content could be reduced to less than 0.02 wt% [6,10]. It is believed that the rubber particles and proteins are both chemically and physically bonded. The chemical bonding is degraded by a proteolytic enzyme, whereas the physical linking is inhibited with urea. As almost all proteins in NR can be removed with urea, it is assumed that most proteins in the NR latex interact with the rubber particles through physical interactions [6,10]. Recently, the influence of proteins on various properties of raw NR and its vulcanizate has been investigated [3]. It was found that removing the protein resulted in a significant loss in the mechanical characteristics of NR in its not vulcanized stage due to the destruction of a pseudo-network formed by the protein and other non-rubber components. Due to the more rigid network linkages by sulfur chemical bonds, the changes in these transient weak networks had only a minor effect on the mechanical properties of the vulcanized rubber.

Although these investigations yielded much important information, the effects of incubation and washing/centrifugation remain not well understood, although their combined effects have been studied. To clearly understand the changes in microstructure during deproteinization stages, the influences of incubation and washing/centrifugation on the physical, mechanical, and dynamic properties of NR latex were assessed in the current study. Finally, the effects of incubation and washing/centrifugation were distinguished, and a model explaining the destruction of the natural network formation during each step of deproteinization is proposed.

## 2. Materials and Methods

### 2.1. Materials

Commercial high ammonia natural rubber (HANR) latex was bought from the Yala Latex Industry (Yala, Thailand). The HANR latex had approximately 61.5% total solids content and 60.1% dry rubber content (drc).

### 2.2. Preparation of DPNR

**Figure 1:**
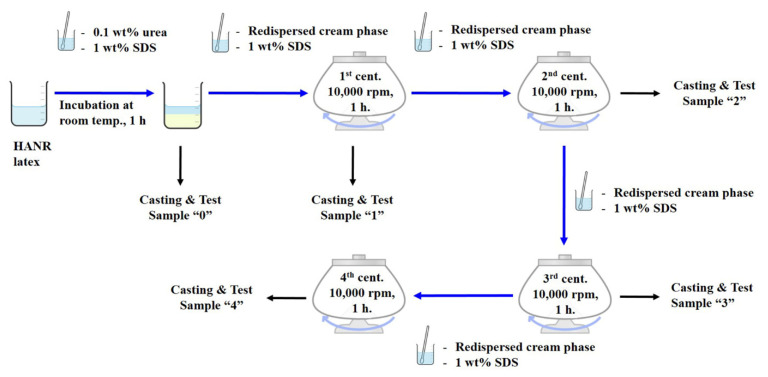
Preparation of DPNR with multiple washing/centrifugation cycles at 12,320 *g*-forces.

The DPNRs were prepared according to a procedure reported previously [6]. As shown in Figure 1, the HANR latex was incubated with 0.1 wt% urea and 1 wt% sodium dodecyl sulfate (SDS) at 30 °C for 1 h. The cream fraction was re-dispersed in a 1 wt % SDS solution to make a 30 wt % drc latex before casting. This incubated sample without centrifugation was labeled with “0”. DPNR with one cycle of centrifugation (sample “1”) was produced similarly to sample 0, except that the cream fraction was re-dispersed and centrifuged at 10,000 revolutions per minute (rpm) for 1 h (about 12,320 g-forces). The top layer was re-dispersed in distilled water and cast. The samples labeled “2”, “3”, and “4” were produced similarly to sample 1, except that the cream fraction was re-dispersed and re-centrifuged for 2, 3, or 4 cycles.

### 2.3. Total Nitrogen Content Analysis 

The Kjeldahl method was used to quantify the nitrogen content in the DPNRs with and without washing/centrifugations [11]. Before digestion with concentrated sulfuric acid, a 0.1 g rubber sample was loaded in a Kjeldahl flask containing the Kjeldahl catalyst. After digesting the mixture, 50 mL of sodium hydroxide solution was added. After distilling the solution, the distillate was collected in a boric acid solution. Finally, the ammonia level was measured by titrating the distillate with 0.01 N sulfuric acid. The total nitrogen content was calculated using Equation (1):

(1) $$\%\;\mathrm{Nitrogen}=\frac{2\;\times \;A\;\times \;N\;\times \;14}{1000\;\times \;B\;}\times 100$$

where A is ml of sulfuric acid required for titration, N refers to concentration of standardized sulfuric acid (normality) and B is the mass of the sample (g).

### 2.4. Plasticity Test 

A Plastimeter H-01 (Montech-T, CG Engineering, Pathum Thani, Thailand) was used to measure the plasticity (P_0_) and plasticity retention index (PRI) of the obtained DPNRs. The PRI can be calculated using the following equation.

(2) $$\mathrm{PRI}=\left(\frac{P_{30}}{P_{0}}\right)\times 100$$

where P_0_ represents the initial plasticity, while P_30_ represents the plasticity after 30 min of aging at 140 °C. 

### 2.5. Mooney Viscosity Test 

A Mooney viscometer (MV 3000 Basic, MonTech, Buchen, Germany) was used to investigate the Mooney viscosity of various DPNR samples. The experiment was carried out using the large rotor at 100 °C according to ASTM D1646.

### 2.6. Tensile Property Test 

The tensile properties of all DPNR samples were determined using a universal testing machine, model LR5K plus (LR5K Plus, LLOYD Instruments, West Sussex, UK), with a 500 mm.min^−1^ crosshead speed. The tensile test specimens were prepared in accordance with ISO 37.

### 2.7. Dynamic Mechanical Analysis (DMA) 

The dynamic properties of DPNRs in tension mode were evaluated using a dynamic mechanical thermal analyzer (Eplexor 9, NETZSCH GABO Instrument GmbH, Ahlden, Germany). The DMA was carried out over the temperature range from −80 °C to 80 °C, with a heating rate of 2 °C min^−1^ and a frequency of 10 Hz.

To further understand the effects of wash/centrifugation cycles on natural network chain density, the crosslink density (ν) and the molecular weight between crosslinks (M*_c_*) of not vulcanized DPNRs were determined. The *ν* was estimated by using the following equation [12]:

(3) $$\nu =\frac{E^{\prime }}{6RT}$$

where $E^{\prime }$ is the elastic storage modulus of the rubber, R is the universal gas constant, and T is the absolute temperature. 

The M*_c_* was estimated using Equation (4) [13]:

(4) $$M_{c}=\frac{3\rho RT}{E^{\prime }}$$

where ρ is the rubber density.

## 3. Results and Discussion 

### 3.1. Total Nitrogen Content 

**Figure 2:**
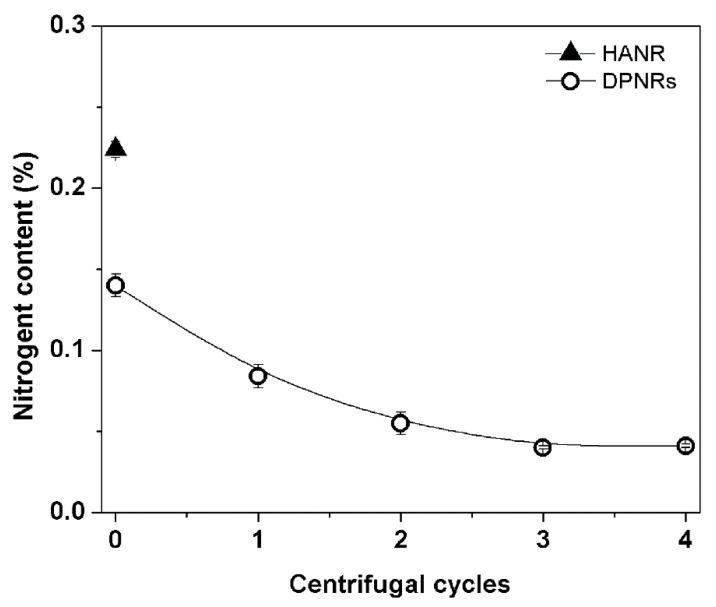
Nitrogen content of HANR and DPNRs.

Figure 2 depicts the effects of incubation and centrifugation on the nitrogen content in NR, indicative of the protein content. As a baseline reference, the initial nitrogen value of concentrated latex (HANR) was also recorded. It has been reported that fresh NR had about 0.58 wt% nitrogen content [9]. Centrifugation is practically employed in converting fresh field latex to HANR. After the centrifugation, the nitrogen concentration of HANR was reduced to 0.224 wt%. This is because the high-speed centrifuging can separate the soluble ingredients, resulting in a significant reduction in the amount of nitrogen in the HANR [14,15,16]. From Figure 2, it is also observed that the nitrogen content had decreased from 0.224 wt% to 0.140 wt% (by about 37.5%) after incubation with 0.1 wt% urea. This nitrogen content further decreased to 0.084 wt% (to about 62.5%) after treatment with high-speed centrifugation (12,320 g-forces). The decrease in nitrogen was more noticeable as the number of washing/centrifugation cycles increased. The maximum nitrogen reduction of about 82.0% was achieved with three cycles of centrifugation. However, longer centrifugation did not alter the nitrogen content. It is believed that only the protein links were destroyed during incubation due to no shear forces being applied in this step. The findings indicate that both incubation and washing/centrifugation are required to reduce the protein content in NR latex successfully. For the preparation of DPNR latex, two cycles of washing were found to be the most effective, as further reductions of nitrogen content were not significant after two cycles of washing/centrifugation.

### 3.2. Plasticity 

**Figure 3:**
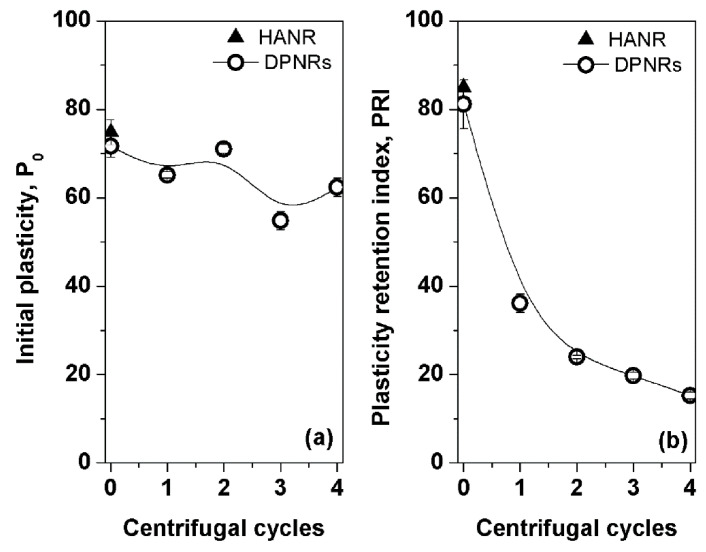
(**a**) Initial plasticity and (**b**) plasticity retention index of HANR and DPNRs.

Figure 3 shows the effect of wash cycles on P_0_ and PRI of the DPNRs. To differentiate the influences of incubation and washes, the P_0_ and PRI values of HANR were also included. It can be seen that removing the proteins resulted in reductions in P_0_ and PRI. This was attributed to the deproteinization causing decomposition of naturally linked network structures induced by proteins. The proteins in the NR play an essential role in the formation of polymer networks because the proteins located at the chain ends of NR can create networks through hydrogen bonding among the terminals [17,18,19]. It is interesting to consider the P_0_ and PRI results of neat HANR and DPNR without centrifugation (sample 0). It was found that the P_0_ and PRI of the HANR were only slightly changed by incubation of the latex with urea, although the nitrogen value was much reduced (see Figure 2). The loss of network linking structures induced by proteins did not much affect the P_0_ and PRI of sample 0. This indicates that the proteins are not the primary source of chain crosslink formation in the NR, as the author has suggested [20]. For this reason, it can be assumed that the natural links present in the not vulcanized NR have several sources. Links are generated by proteins and other impurities such as phospholipids and by the entanglement of rubber polymeric chains. It is assumed that two types of entanglements could possibly be formed in the rubber: chain entanglement and cohesion chain entanglement, as previously found in other polymers [21]. The entanglements between rubber chains must be considered because the lengthy polymer chains are easily entangled to generate tie points. The entangled chains behave just like those with crosslinks, preventing the polymer chains from sliding against each other under load [22]. This could be why the P_0_ and PRI of sample 0 remained high after deproteinizing by incubation with urea solution. In addition to the protein reduction, these entangled tie points were also loosened when the rubber latex was subjected to washing and high-speed centrifugation. Consequently, repeat wash/centrifugation cycles further reduced the natural networks, destroying all network structures arising from proteins, other impurities, and chain entanglements. Therefore, the P_0_ and PRI of the DPNRs were decreased after the deproteinization stages.

### 3.3. Mooney Viscosity 

**Figure 4:**
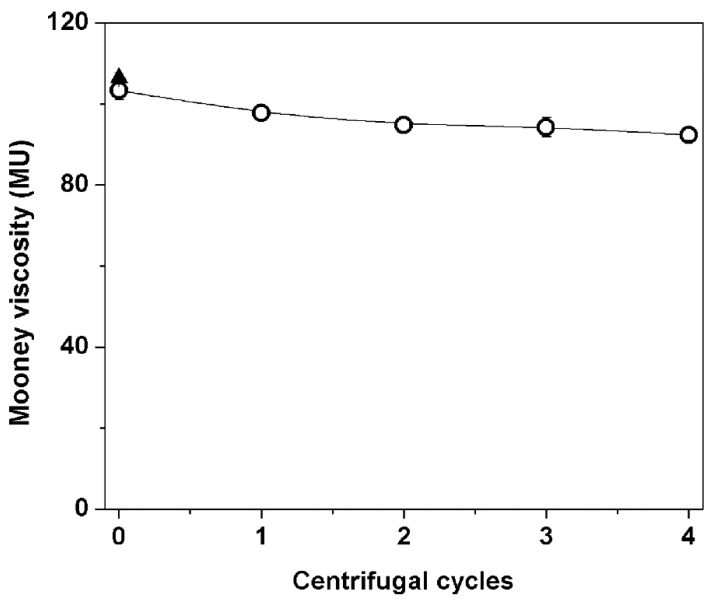
Mooney viscosity of HANR and DPNRs.

The effects of incubation and wash/centrifugation treatments on Mooney viscosity (MV) of the DPNRs are shown in Figure 4. For comparison, HANR’s MV is included. It is seen that the MV of DPNR samples decreased from 106 to 93 MU (Mooney units) as the number of centrifugation cycles increased. The decrease in Mooney viscosity could be attributed to the disintegration of the crosslinked networks in the NR caused by protein removal and the loss of entangled points. The influence of washing with high-speed centrifugation on the viscosity can be assessed by considering the viscosity of pure HANR. After incubation (without centrifugation), the viscosity of HANR was slightly reduced because a portion of the networks induced by proteins was destroyed. Although the linkages that originated from proteins were broken, the network links by polymer entanglements remained. Thus, the viscosity of the incubated sample was almost comparable with the viscosity of HANR. Such evidence highlights the importance of entanglements as network linkages. It should be noted here that a reduction in MV was found with wash/centrifugation cycles, but the MV in all cases remained considerably high. The cause for this is most likely the large molecular weight of NR, as the cycles did not affect its molecular weight [3,18]. 

**Figure 5:**
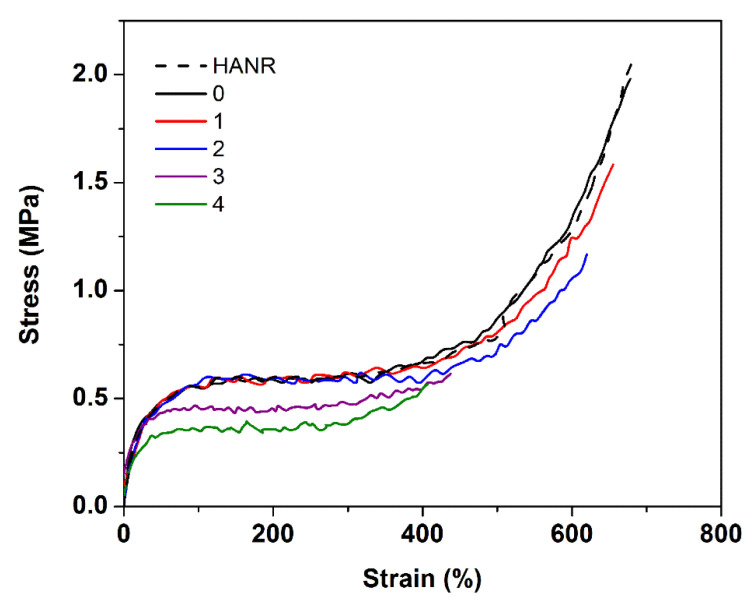
Representative stress-strain curves of HANR and DPNRs.

Figure 5 shows stress-strain curves of the HANR and DPNRs with different wash/centrifugation cycle counts. Because of the contribution from natural networks in the NR, the green tensile strength of the HANR was the highest. As previously mentioned, these networks use several links generated by proteins and other impurities, such as phospholipids and polymer chain entanglements. The tensile strength and elongation at the break of the HANR were slightly reduced after incubation of the latex, caused by the loss of some networks associated with proteins. When the incubated latex was subjected to washing and high-speed centrifugation cycles, the tensile strength and extensibility decreased accordingly, consistently with the cycle count. A significant reduction in stress at various strains, tensile strength, and the tensile maximum strain was noticed for the DPNRs subjected to more than two wash cycles (see samples 3 and 4). This is associated with a significant decrease in protein content and the breakdown of all networks in the DPNR. 

Self-reinforcement can be seen in the steep increase in stress at the high strain in the stress-strain curve, and this pattern was almost completely lost after two cycles of washing/centrifugation. The sharp upturn in stress-strain curves has long been assumed to be caused by strain-induced crystallization during stretching [23,24,25]. The short chains between links in networks were firstly stretched, and the fully stretched network chains then acted as starting points of strain-induced crystallization during deformation. The crystallites then grow and act as a new crosslink site or hard filler, offering self-reinforcement of the rubber with hardening [26,27,28,29]. Thus, the tensile stress steeply increased with strain-induced crystallization. The steeply increasing stress in NR, therefore, indicates the presence of natural networks in the rubber, whereas the absence of such networks is suggested by the lack of a sharp stress increase. Similar behavior has been reported earlier [3]. The results clearly confirm that the networks in the incubated NR latex were drastically destroyed by washing and centrifugation for more than two cycles. 

**Figure 6:**
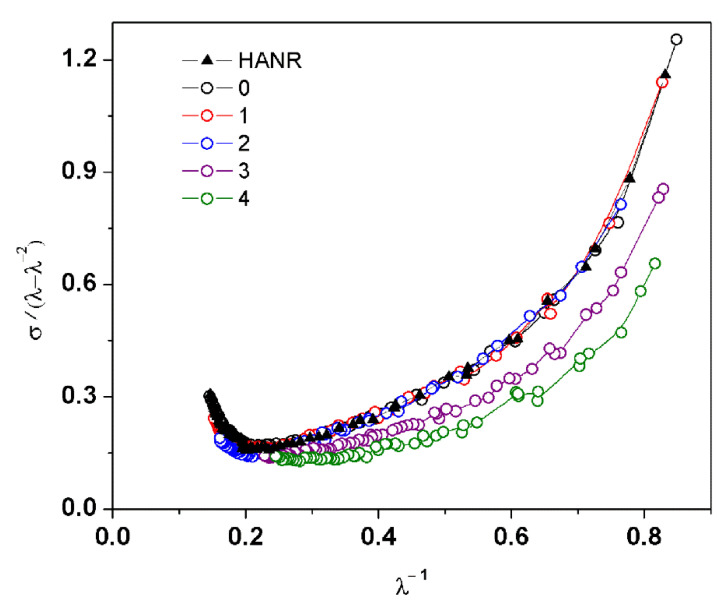
Mooney–Rivlin plots for HANR and DPNRs.

To acquire more details of reinforcement, the stress-strain curves of all samples were assessed in Mooney–Rivlin plots, see Figure 6.

It can be observed that the samples without and with a small number (one or two) of washing and centrifugation cycles showed a steeper upturn of σ/(λ–λ^−2^) at low 1/λ. It is well-known that such a steep upturn reflects the strain-induced crystallization in NR [30]. As the wash cycles were repeated, the strain-induced crystallization diminished due to the destruction of all-natural networks. Therefore, it can be concluded that deproteinization and washing/centrifugation for more than two cycles drastically reduced the network linkages in the NR.

### 3.4. Tensile Properties 

**Figure 7:**
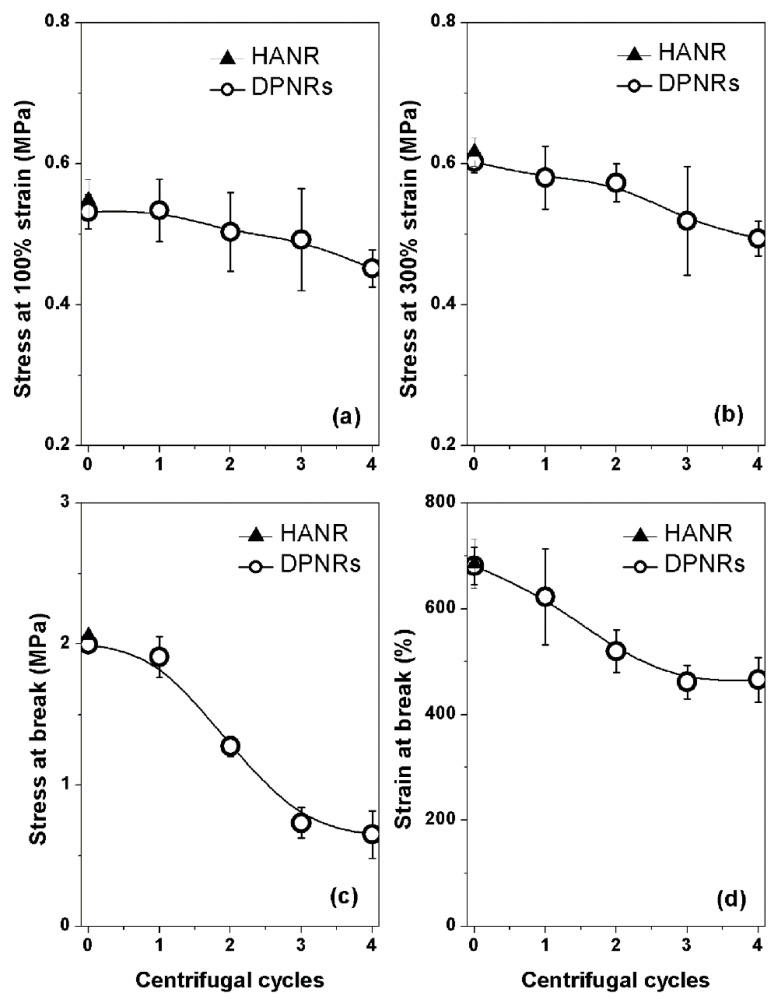
(**a**) stress at 100% and (**b**) 300% strains, (**c**) stress at break, and (**d**) strain at break of HANR and the DPNRs.

The values of stress at 100% and 300% strains, stress at break, and strain at break from the tensile tests of DPNRs are presented in Figure 7a–d. The strain at the break of the incubated latex (sample 0) was slightly lower than that of the HANR due to damage to some networks that had been created by the proteins. In contrast, other linkages by impurities and entanglements remained. All tensile properties deteriorated with increasing count of washing and centrifugation cycles due to the loosening of all links in the NR networks.

### 3.5. Dynamic Mechanical Property 

**Figure 8:**
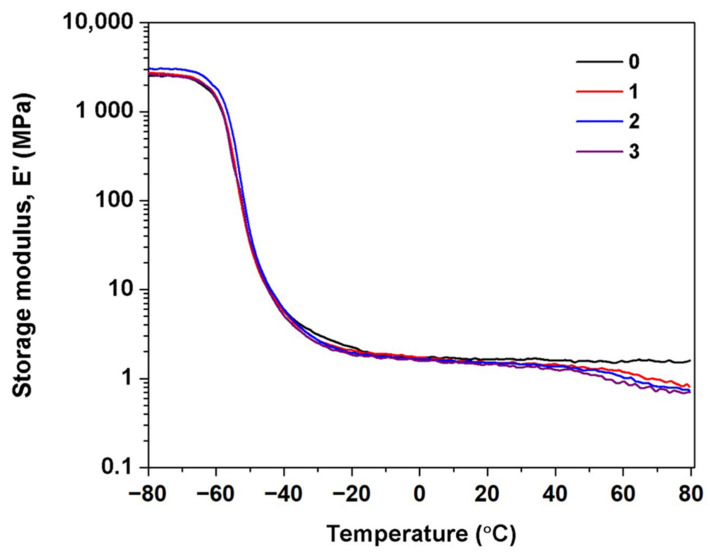
Storage module (*E*^′^) as a function temperature for DPNRs with different counts of centrifugation cycles.

To further inspect the effects of washing and centrifugation on the dynamic mechanical properties of DPNRs, the DMA measurements were done on DPNRs without and with 1 to 3 cycles of washing. Since the mechanical properties of HANR were not drastically different from the DPNR without centrifugation, it is assumed that also the dynamic mechanical properties of these two samples differ negligibly. Figure 8 displays the storage modulus (E’) for the DPNRs with and without washes as a function of temperature in the range from −80 to 80 °C. Details of E′ in the rubbery plateau region are summarized in Table 1. Focusing on the modulus at the rubbery plateau region, E′ decreased with wash cycles from 1.60 to 1.33 MPa, indicating that rubber materials’ stiffness and hardness were reduced. This was probably due to the loss of linkages between the rubber chains with increasing centrifugation cycle count, explaining decreased modulus and strength of the materials. It is noted that the modulus of centrifuged DPNRs decreases sharply when the temperature rises over 40 °C, depending on the count of wash cycles. This reduction is tentatively attributed to the degradation of branch points originating from interactions between phospholipids and α-terminal groups of the rubber molecules [31]. Besides, disentanglement among the rubber molecular chains could also substantially reduce the modulus of DPNRs. This is attributed to most of the physical linking points deteriorating at elevated temperatures. Therefore, the repeated washing and centrifugation cycles drastically reduced linkages from proteins and entangled chains, contributing to network formation. Thus, the reduction of modulus kept accumulating with washing and centrifugation cycles.

**Figure 9:**
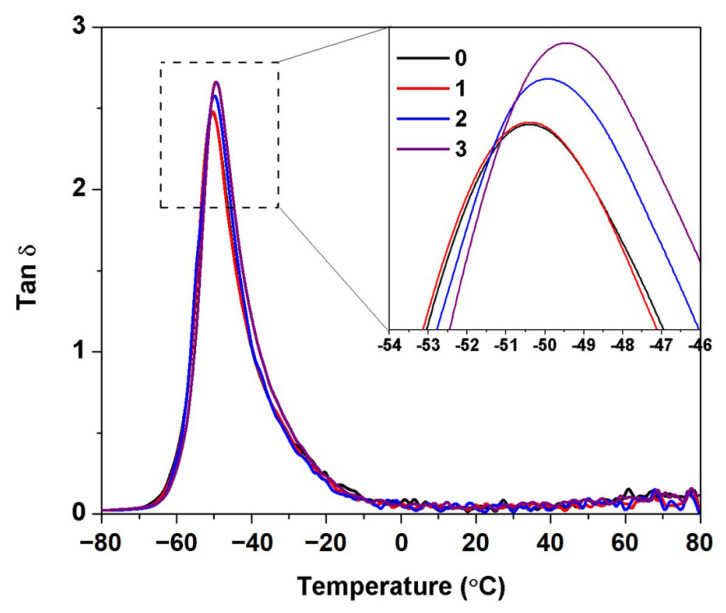
Tan δ as a function temperature for DPNRs with different counts of centrifugation cycles.

Figure 9 shows the loss factor (Tan δ) of the DPNRs with and without wash cycles. The glass transition temperatures (Tg) and maximum Tan δ peak heights are summarized in Table 1. Tan δ is defined as the ratio of loss modulus to storage modulus. It is used to measure the energy loss and reflects the mechanical damping by molecular internal friction of a viscoelastic material. As the number of was cycles increased, the tan δ peak corresponding to Tg slightly moved towards higher temperatures, i.e., from −50.42 to −48.42 °C. The loss of links from networks can explain this behavior. Without the linkages, the mobility of rubber chains increased, but the molecular chain’s flexibility decreased. Moreover, the Tan δ peak height (Tan δmax) of DPNRs also increased with the wash cycles. The loss of chain linkages increases the flowability of the material, giving rise to energy losses within the material during viscous deformation. As a result, the Tan δ peak height increases.

It is universally acknowledged that the *E*^′^ at the rubbery plateau region can be utilized to estimate the crosslink density (*ν*) and the molecular weight between crosslinks (M*_c_*) [13]. Table 1 summarizes *ν* and M*_c_* for the various DPNRs. It can be seen that *ν* decreased with washing/centrifugation cycles, revealing that this processing broke down linkages originating from proteins, other non-rubber components, and entangled chains. The wash/centrifugation cycles increased M*_c_* as linkages by entanglement were destroyed. Since the protein was located at the polymer chain end [17], the loss of links generated by proteins should indeed not affect the M*_c_* of rubber.

**Figure 10:**
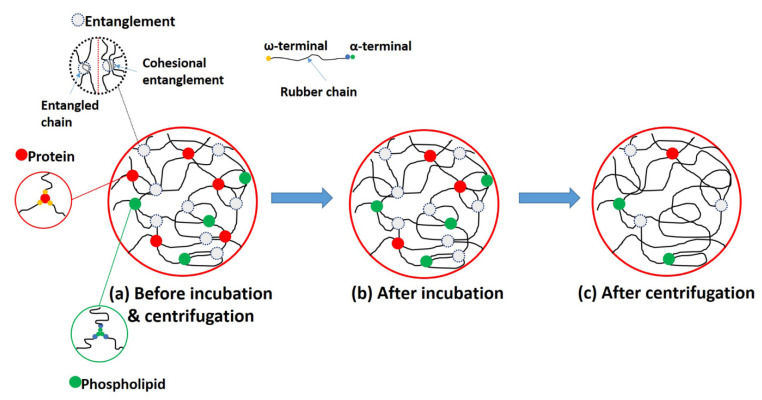
Proposed model for latex before and after the deproteinized process.

Based on the findings of this study, a mechanistic model of network link destruction in the DPNRs was proposed, as illustrated in Figure 10. In this model, the dried film from latex before incubation and centrifugation contained several linkages attributed to proteins, phospholipids, and entangled chains (Figure 10a). The α-terminated chain is linked to phospholipids and the ω-terminated chain to proteins via H-bonding [17,32]. In the case of entangled chains, there are two possible forms of entanglement: chain entanglement and cohesion chain entanglement. All these links appear naturally in networks in the NR. When the latex was treated with urea, part of the proteins was eliminated due to them being cut loose from the rubber particles, and this left fewer protein linkages (Figure 10b). Although the proteins that formed the linkages were destroyed, the rubber still had other remaining links that provided strength to the rubber, so the strength and other properties changed only slightly. Depending on the count of washing/centrifugation cycles, the residual linkages were eliminated from the incubated latex (Figure 10c). High-speed centrifugation could well have disrupted the entanglements of the cohesive chains. Therefore, the mechanical characteristics of rubber substantially deteriorated after washing/centrifugation of the incubated latex.

## 4. Conclusions

Influences of incubation and washing/centrifugation cycles for deproteinization on the properties of DPNRs were investigated. The results revealed that proteins, phospholipids, and chain entanglements were responsible for natural network formation in the NR. Although proteins were largely removed from the latex by incubation with urea, the mechanical characteristics of plasticity, viscosity, and tensile strength persisted with little change in DPNR, implying that other network linkage types dominate over the protein linkages. The performance attributes of DPNRs were dramatically diminished by washing/centrifugation, depending on the number of such cycles. As the number of centrifugation cycles increased, the number of network links in the DPNR decreased. This resulted in a drastic loss of mechanical characteristics. With repeated washing/centrifugation cycles, the storage modulus and the chain flexibility were found to decrease. Two cycles of washing/centrifugation appeared the most appropriate choice, not yet substantially degrading the mechanical properties of DPNR, but leaving little protein that could be allergenic in common applications.

## Figures and Tables

**Table 1:** Values of *E*^′^, T_g_, Tan δ_max_, *ν*, and M_c_ of DPNRs.

Centrifugation Cycle	*E*^′^ (MPa)	Tg (°C)	Tan δ_max_	*ν* (moles/m^3^)	M_c_ (g/mol)
0	1.60	−50.42	2.50	105.24	4371
1	1.46	−50.42	2.50	96.10	4786
2	1.44	−49.25	2.59	94.64	4860
3	1.33	−48.42	2.68	87.87	5235

## Data Availability

The data presented in this study are available on request from the corresponding author.
